# The Influence of Co-action on a Simple Attention Task: A Shift Back to the Status Quo

**DOI:** 10.3389/fpsyg.2018.00874

**Published:** 2018-06-04

**Authors:** Jill A. Dosso, Kevin H. Roberts, Alessandra DiGiacomo, Alan Kingstone

**Affiliations:** Department of Psychology, University of British Columbia, Vancouver, BC, Canada

**Keywords:** line bisection, social presence, replication, joint attention, joint action, covert attention

## Abstract

There is a growing consensus among researchers that a complete description of human attention and action should include information about how these processes are informed by social context. When we actively engage in co-action with others, there are characteristic changes in action kinematics, reaction time, search behavior, as well as other processes (see Sebanz et al., 2003; Becchio et al., 2010; Wahn et al., 2017). It is now important to identify precisely what is shared between co-actors in these joint action situations. One group recently found that participants seem to withdraw their attention away from a partner and toward themselves when co-engaged in a line bisection judgment task (Szpak et al., 2016). This effect runs counter to the typical finding that attention is drawn toward social items in the environment (Birmingham et al., 2008, 2009; Foulsham et al., 2011). As such, the result suggests that joint action can uniquely lead to the withdrawal of covert attention in a manner detectable by a line bisection task performed on a computer screen. This task could therefore act as a simple and elegant measure of interpersonal effects on attention within particular pairs of participants. For this reason, the present work attempted to replicate and extend the finding that attention, as measured by a line-bisection task, is withdrawn away from nearby co-actors. Overall our study found no evidence of social modulation of covert attention. This suggests that the line bisection task may not be sensitive enough to reliably measure interpersonal attention effects – at least when one looks at overall group performance. However, our data also hint at the possibility that the effect of nearby others on the distribution of attention may be modulated by individual differences.

## Introduction

By its very nature, spatial attention involves the selection of some locations or objects rather than others. This is readily seen when the normal operation of attention breaks down, as in the case of patients with unilateral spatial neglect. Such patients experience pathological disruptions to their spatial attention as a function of right parietal lobe damage. This damage results in biased attention to rightward locations and objects at the expense of attention to leftward locations and objects (Corbetta and Shulman, 2011; Karnath, 2015). Even in the typical population, however, there is evidence of asymmetries in spatial attention. Reliably, typically developing individuals allocate slightly more attention to the left side of space. This small bias to overestimate or over-attend the left side of space can be seen in the overestimation of the length of felt and imagined lines (Brooks et al., 2014), in the greater tendency to miss rightward items when left and right locations are stimulated simultaneously (Goodbourn and Holcombe, 2015), in spontaneous looking behavior (Nuthmann and Matthias, 2014), and perhaps most routinely, in the standard visual line bisection task (Jewell and McCourt, 2000).

In the prototypical line bisection task, participants are asked to judge whether a mark (“transector”) on a long horizontal line is located to the right or to the left of the horizontal line’s true center. Typically, on-screen cues that precede the presentation of the line have been shown to attract attention, inducing a perceived lengthening of the line segment nearest the cue (McCourt et al., 2005; Toba et al., 2011). Importantly, one study found that distractors could influence line bisection performance without being fixated. Covert attention, therefore, is sufficient to produce these effects (Thomas et al., 2015).

Recently, the notion that social stimuli could induce these same types of attention shifts has been investigated. In non-bisection tasks, gazing eyes have been shown to reflexively bias attention in the direction of their gaze (Friesen and Kingstone, 1998; Kuhn et al., 2009), even among patients with left-neglect (Bonato et al., 2008). Moreover, social stimuli including the eyes are preferentially looked at when images are viewed (Birmingham et al., 2008, 2009; Foulsham et al., 2011); and when a visible experimenter was used as a distractor in a line bisection task, a perceptual-attentional bias in line bisection toward the experimenter was documented (Garza et al., 2008). Thus, the consensus across a large body of work is that attention shifted by and toward social information within a scene (Friesen and Kingstone, 1998; Driver et al., 1999; Vuilleumier, 2000; Kuhn and Land, 2006; Theeuwes and Van der Stigchel, 2006; Birmingham et al., 2009; Laidlaw et al., 2012; Rösler et al., 2017).

It is unclear, however, whether the presence of co-actors will also shift attention in a similar manner (Hayward et al., 2017). Commonly, joint action studies feature two individuals facing and acting together on stimuli presented on a computer screen (e.g., Sebanz et al., 2003; Eskenazi et al., 2013; Brennan and Enns, 2015; Dudarev and Hassin, 2016; Wahn et al., 2017). Employing the line bisection task in this format could therefore provide a simple index of the co-actor’s impact on the topography of attention within the screen. Recent papers have found evidence that, in contrast to the large body of evidence touched on above, attention is directed away from live co-actors, inducing a perceived shortening of the line segment nearest the other person (i) in the horizontal plane when pairs of individuals sit facing the same direction, and (ii) in the radial direction when individuals face one another (albeit only among those who show a high level of physiological arousal) (Szpak et al., 2015, 2016). This unique finding that, in some cases, joint action can lead to changes in the static topography of covert on-screen attention is surprising because it suggests that live co-actors impact attention quite differently than one would expect, given the established literature. In addition, this effect seems to extend beyond the physical body of the co-actor, and to include the jointly attended computer monitor. This task could therefore act as a simple and elegant measure of interpersonal effects on attention within particular pairs of participants.

There are, however, two outstanding points regarding this measure. First, the attentional withdrawal effect appears to be quite small. Social Influence Score (SIS) – the index of attentional attraction or withdrawal that was used – was calculated as a value in millimeters across three experiments (Szpak et al., 2016). This value was obtained by comparing the perceived midpoint of horizontal lines when seated beside a co-actor versus when seated alone. A shift in the perceived midpoint toward the co-actor (positive SIS) was taken as evidence of attentional attraction, whereas a shift away from the co-actor (negative SIS) was taken as evidence of attentional withdrawal. SIS had a negative value in all three experiments, consistent with attentional withdrawal away from the co-actor, but this value was significantly different from zero (i.e., no change in attention) only for two of three experiments. Moreover, the three SIS values were not different from one another across the three experiments, rendering any conclusions to be of an equivocal nature. Thus, it seemed valuable to replicate the effect in a different laboratory to assess its reliability. Second, though Szpak and colleagues report the attentional withdrawal effect at a group level, the significance of the effect across individuals, and its relationship to individual and pair factors, is not yet known.

Given the potential value of the paradigm regarding social attention, the present work sought to replicate the reported bias in horizontal line bisection away from nearby others, and to form an exploratory profile of potential individual differences in the population in the extent to which they show an effect (Szpak et al., 2016). Based on Szpak and colleagues attentional withdrawal hypothesis, one would predict that participants will overestimate the length of the line segment nearest themselves to a larger extent when in the presence of a partner rather than when alone. On the other hand, if the partner draws attention in the same way as other cue types, one would expect participants to instead overestimate the length of the more distant line segment (Toba et al., 2011) to larger extent in the direction of the partner’s location. A third possibility given the possible marginal magnitude of the effect is that nearby others may have no impact on attention in this context, from which one would predict no significant shift in line bisection performance across manipulations of partner position.

## Materials and Methods

Sample size was selected based on an *a priori* power analysis using G^∗^Power 3.1.9.2 software (Faul et al., 2007). In their first experiment, Szpak et al. (2016) report as their main measure of interest a SIS of -0.22 mm (*SD* = 0.43) and this was compared to a theoretical value of zero (which would indicate that attention is neither attracted nor withdrawn from the co-actor). An effect size (*d*) was calculated to have a value of 0.51. In order to detect this effect with a power of 0.80, a total sample size of 16 pairs (32 participants) was required. More participants than this were collected in anticipation of the need to make exclusions. Participants were recruited from a pool of undergraduate students and received course credit for participating. Twenty-seven pairs (*n* = 54) were tested. Mean age was 21.4 years (*SD* = 5.2). Participants self-identified as female (*n* = 42) and male (*n* = 12). Their self-reported ethnicities were Asian (*n* = 35), Caucasian/White (*n* = 14), Latin American (*n* = 1), Middle Eastern (*n* = 1), Multiethnic (*n* = 1), and undisclosed/could not be categorized (*n* = 2). Based on their handedness responses (Oldfield, 1971), they were right-handed (*n* = 51) or ambidextrous (*n* = 3). Participants were paired with one another at random, and provided informed consent before participating.

**FIGURE 1:**
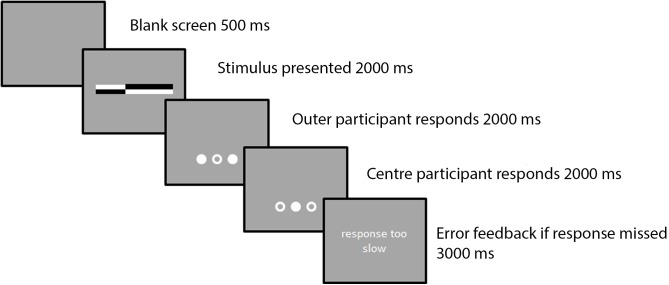
A sample trial sequence.

Three chinrests were placed 450 mm apart. Stimuli were created and presented using PsychoPy software (Peirce, 2007). Each black and white line was 18 mm long, and was bisected at one of six possible locations (-3, -2, -1, 1, 2, or 3 cm from true center, see **Figure 1**). The central chinrest was located in front of the monitor at a distance of 600 mm, within peripersonal space (Gamberini et al., 2008). On each trial, participants were instructed to indicate, using keypresses, the shorter side of each line^1^. The absolute position of each line was jittered between trials from -1.5 to 1.5 mm of the true center of the screen. Three circles indicated when each participant should provide their response. Participants’ hands were covered by a cloth, preventing them from seeing one another’s responses. These circles were also jittered -1.5 to 1.5 mm from true center. On each trial, the order of participants’ responses was randomized. There were 72 trials per block. Each pair participated in six blocks: one person would be seated in the center for three blocks in which their partner was seated on the left, seated on the right, and absent from the room (these blocks presented in a random order). Then, the procedure was repeated with the other participant seated in the center.

After testing, questionnaire responses were collected: demographic information, ratings of participants’ liking and awareness of their partner, the Inclusion of Other in the Self scale, the Edinburgh Handedness Inventory-short form, the Self-Consciousness Scale, and the Autism Spectrum Quotient (Scheier and Carver, 1985; Aron et al., 1992; Baron-Cohen et al., 2001; Veale, 2014).

**FIGURE 2:**
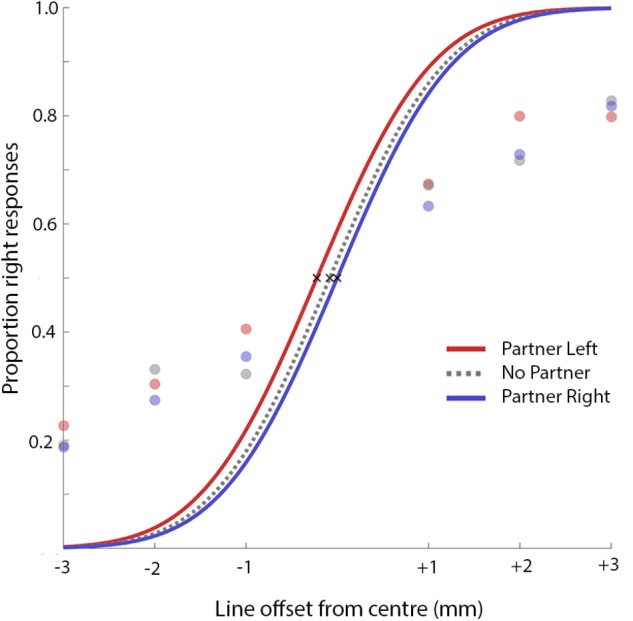
Curves fitted to mean results from all thirty-one participants. Note that this is for illustrative purposes only and does not represent the inferential tests, which were performed on per-participant threshold values.

Data from two pairs were excluded because one member failed to comply with instructions. In addition, three individuals were excluded when testing sessions were forced to end early, one individual was excluded for self-reporting an attention-related diagnosis (ADHD), and three individuals were excluded after reporting that their vision was below normal and uncorrected, but in these cases data from partners was retained in the analysis. Furthermore, 12 additional participants were excluded for responding with more than 90% right or left answers on a single block or who, on any block, made more “right is longer” responses in the most extreme leftward bisection condition as compared to the most extreme rightward bisection condition. This yielded 31 participants in the final analysis. From participants’ responses, the *point of subjective equality* (the theoretical line bisection position for which the participant would produce 50% “left” and 50% “right” responses) was calculated for each block in which they were seated in the center. This procedure was intended to match previous work (Nicholls et al., 2014; Szpak et al., 2016). Line bisection thresholds were estimated separately for each participant and each seating condition (partner left, no partner, partner right) by fitting psychometric functions to response data using the Palamedes toolbox (Prins and Kingdom, 2009). A cumulative Gaussian function was fit to response data using a Maximum Likelihood criterion, where the threshold parameter was free to vary, the slope was fixed at 1, and the guess and lapse rate were both fixed at 0 (**Figure 2**); these parameters are consistent with the function fitting performed in Szpak et al. (2016).

## Results

**FIGURE 3:**
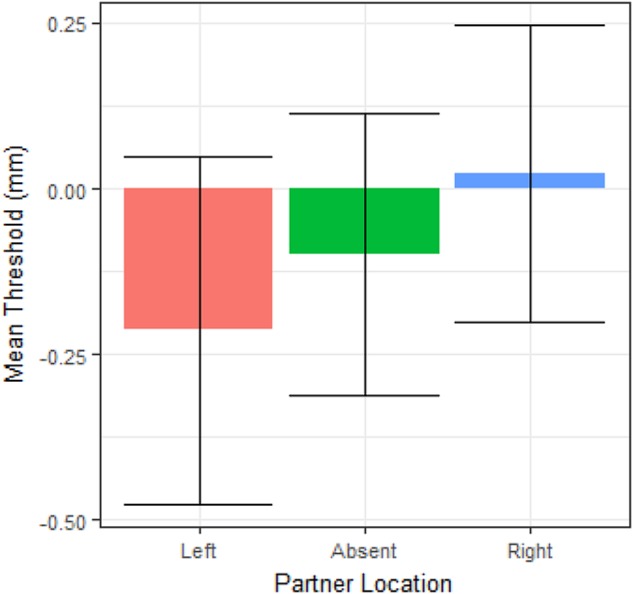
Mean line bisection thresholds across partner locations. Error bars represent within-subject 95% confidence intervals (Cousineau, 2005; Morey, 2008). Note that thresholds are not different based on partner position.

**FIGURE 4:**
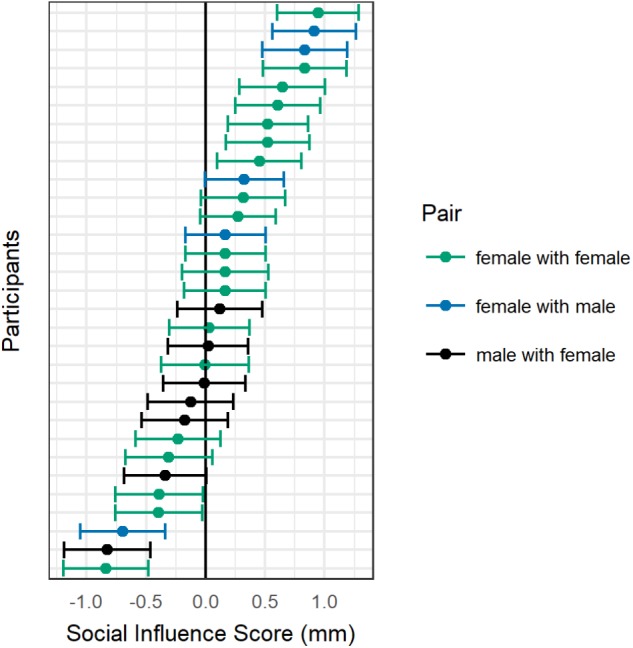
Distribution of attentional shifts across the sample. For each participant, Social Influence Score (SIS) with 95% confidence interval is shown.

All supporting data for this paper are available at https://osf.io/pghe5/. **Figures 3**, **4** were generated using the ggplot2 package in R software (Wickham, 2009).

### Preplanned Analyses

Based on the thresholds identified for each participant for each partner location (**Figures 2**, **3**), the mean change in threshold toward the other individual was calculated in mm, termed the “SIS” (Szpak et al., 2016). A positive SIS indicates a shift in attention toward the other individual while a negative score indicates a shift in attention away (and toward the self). In their first experiment, Szpak and colleagues found a mean SIS of -0.22 mm which was significantly different from zero. In the current study, mean SIS was found to be 0.12 mm, with a 95% confidence interval of (-0.06, 0.30). A Bayes Factor for this analysis was obtained using the ttestBF function in the BayesFactor package for R (Morey and Rouder, 2015). Mean SIS was not significantly different from zero [two-tailed, one-sample *t*-test: *t*(30) = 1.35, *p* = 0.19; BF_10_ = 0.44]. A Bayes Factor smaller than one indicates greater evidence for the null hypothesis (the measured value is not different from zero) than the alternative hypothesis (the measured value is different from zero). In this case, the data are 1/0.44 or 2.3 times more likely under the null than the alternative hypothesis. However, the present mean SIS was significantly different from that calculated by Szpak and colleagues [two-tailed, one-sample *t*-test: *t*(30) = 3.87, *p* = 0.0006, BF_10_ = 55.7]. A Bayes Factor between 10 and 100 is considered “strong” evidence for the alternative hypothesis that our measured value is different than the comparison value (Kass and Raftery, 1995).

### Exploratory Analyses

In the original work by Szpak and colleagues, calculation of SIS involved collapsing effects across left and right seating positions. To investigate the possibility that leftward and rightward effects might differ in our sample, a within-subjects ANOVA was performed with partner location (left, right, and absent) as the IV and threshold as the DV. This analysis revealed no effect of partner location on line bisection thresholds [*F*(2,60) = 1.07, *p* = 0.35]. To evaluate whether this constituted good evidence for the null hypothesis, a Bayesian ANOVA was performed using the BayesFactor package in R (Morey and Rouder, 2015). The Bayes Factor (BF_10_) for this analysis was 0.13. Therefore, these data are 1/0.13 or 7.7 times more likely under the null hypothesis than under the alternative hypothesis.

To address the question of whether individual participants could show meaningful shifts toward or away from their partner, the error around each participant’s individual SIS was calculated (**Figure 4**). To estimate the error of threshold estimates, a non-parametric bootstrap was performed, using 1000 bootstrap simulations for each condition. The standard error for the SIS for each participant was calculated as the standard deviation of the composite bootstrapped sampling distribution created by averaging the subtraction of the “no partner” from the “partner right” and the “partner left” from the “no partner” bootstrapped sampling distributions. The 95% CI was calculated individually for each participant as their SIS estimate, ±1.96 times the standard error. A negative SIS with a 95% CI that did not include zero was considered attentional withdrawal for that individual. A positive SIS with a 95% CI that did not include zero was considered attentional attraction for that individual. Within the final sample of 31 participants, five instances of attentional withdrawal were found (three females paired with females, one female paired with a male, one male paired with a female) and nine instances of attentional attraction (seven females paired with females, two females paired with males). The remaining 17 individuals in the sample did not fit either definition and could be considered attentionally neutral with respect to their co-actor. To investigate potential sources of this individual difference in SIS, the correlation between SIS and the following measures was calculated: rating of liking the partner (1–5), rating of awareness of the partner (1–5), self-other integration score, total score on the self-consciousness scale, and total score on the autism quotient. None of these measures was significantly correlated with SIS (all *r* between -0.16 and 0.01, all *p* > 0.42). However, male and female subjects differed from one another in their SISs [*t*(15.6) = 2.57, *p* = 0.02, BF_10_ = 1.58], with women showing positive scores on average (*M* = 0.20) and men showing negative scores on average (*M* = -0.19), see **Figure 3**. Subjects who were tested first within their pair did not significantly differ in SIS from subjects who were tested second [*t*(23.8) = -0.19, *p* = 0.85, BF_10_ = 0.35].

## Discussion

The present work attempted to replicate and extend line-bisection as an effective method for measuring a spatial change in social attention. Previous work found that during a joint line bisection task, on-screen attention was biased away from the side of the screen nearest the co-actor (Szpak et al., 2016). Thus this task could provide a useful and straightforward index of social attentional shifts, and could be used alongside paradigms that measure action kinematics, reaction time, and search behavior in joint contexts (Sebanz et al., 2003; Becchio et al., 2010; Wahn et al., 2017). To further characterize the tool, measures about the individual (Autism-Spectrum Quotient, Self-Consciousness Scale) and the pair (Inclusion of Other in the Self Scale, ratings of awareness and liking of the other individual) were collected in order to try to capture sources of individual differences in this measure. The task was matched to the original paradigm on a host of factors, including stimulus dimensions, viewing distance, interpersonal spacing, and sequence of blocks and of trials. Task instructions differed from those used in the original paradigm but more closely resembled those used in the literature (McCourt and Olafson, 1997; Toba et al., 2011). A control experiment (see footnote 1) excluded instruction as a meaningful source of empirical variation between experiments.

This work failed to replicate the effect of attentional withdrawal from the co-actor as measured by on-screen line bisection performance. These discrepant results suggest three possibilities. First, it may be that the attentional withdrawal phenomenon is real but fragile, such that small cross-laboratory differences or demographic differences between previous and current samples, extinguish the effect at the group level. In this scenario, the present work would represent a false negative with respect to the “true” effect, or would capture a boundary condition under which this effect is not observed. Assuming that the effect size of the original study is accurate, the present failure to replicate is unlikely to be a false negative due to inadequate power due to the combination of an achieved power of 0.79, the observation of a positive overall SIS, and strong evidence that this value differed from that obtained by Szpak et al. (2016). A second possibility, given the discrepancy between current and previous work, is that the attentional withdrawal phenomenon is real but, due to the small power of the original study, the original effect size estimate was inflated and thus the present study was underpowered (Ioannidis, 2008; Button et al., 2013; Open Science Collaboration, 2015). This seems unlikely for the same reasons mentioned above; the two results were significantly different from one another and differ in their direction rather than simply their magnitude. This seems to indicate that the two studies do not capture the same process.

A third possibility is that co-actors do not impact line bisection performance in this paradigm, and prior work reflects an unfortunate false positive.

There are two methodological points that merit consideration here. First, Szpak et al. (2016) do not report details about the fit of their curves. Our participants often failed to reach 100% “left” responses in the leftmost stimulus condition (and 100% “right” in the rightmost, see **Figure 2**), presumably because even the most extreme stimulus conditions remained somewhat difficult. Assuming that the current data resembles the previous sample, this raises a concern about the validity of this procedure as a measure of line bisection thresholds. While the current work followed the procedure used by Szpak et al. (2016) for the purpose of a straightforward replication, future work might employ more sophisticated curve-fitting (e.g., allowing additional parameters in addition to threshold to vary) to ensure that PSE calculations are truly reflective of participants’ response patterns. Second, the present study excluded a number of participants whose data did not meet criteria regarding accurate task performance (12 participants were excluded who responded with more than 90% right or left answers on a single block or who, on any block, made more “right is longer” responses in the most extreme leftward bisection condition as compared to the most extreme rightward bisection condition). Szpak and colleagues report excluding a maximum of three participants per experiment based on the width of their psychometric functions. While it is certainly possible that the original group were able to obtain superior participant compliance through some other means, the discrepancy is notable. If the present data are re-examined to include all participants who were initially excluded for data quality reasons, mean SIS actually takes on a significantly positive value [*M* = 0.24 mm; two-tailed, one-sample *t-*test: *t*(42) = 2.06, *p* = 0.046, BF_10_ = 1.11]. Thus, the inclusion of additional participants does not lead to a replication of the attentional withdrawal effect obtained by Szpak and colleagues; if anything, it provides support for an attentional attraction effect that dovetails with much of the social attention literature (e.g., Toba et al., 2011).

While evidence of attentional withdrawal in the joint line bisection task was not shown at the group level, exploratory analyses revealed an interesting underlying structure within the current sample. First, a subset of individuals showed evidence of attentional withdrawal (16%) while others showed attentional attraction (29%). As noted, attentional attraction is consistent with the task performance one would expect based on the bulk of the social attention and line bisection literatures (Friesen and Kingstone, 1998; Theeuwes and Van der Stigchel, 2006; Garza et al., 2008; Toba et al., 2011), suggesting that for these participants, the co-actor might impact the attention system through similar mechanisms as those involved for other cue types. Attentional withdrawal, on the other hand, is consistent with the social discomfort hypothesis: that attention is withdrawn from nearby others under conditions of personal space invasion (Terry and Lower, 1979; Szpak et al., 2016). None of the questionnaire measures correlated with the SIS, so it is difficult to speculate about any underlying dimensions on which participants varied that could explain their different performances: self-consciousness, autistic traits, integration of the other into the self, and awareness or liking of the other individual were all independent of SIS. However, gender emerged as an organizing variable, with men generally showing attentional withdrawal from the co-actor, and women showing attentional attraction. It would be interesting to investigate in the future whether men experienced the situation as more invasive of their personal space (as would be predicted by the social discomfort hypothesis), perhaps due to larger body size, and/or whether women were more likely to attend to the other individual as they would other cue types (as would be predicted by the majority of the line bisection literature). The latter prediction could be consistent with work finding differences in sensitivity to social information across the sexes. This includes a higher willingness to make eye contact and a stronger tendency to orient to faces by female as compared to male infants, and stronger gaze-cueing effects in female as compared to male adults (Connellan et al., 2000; Lutchmaya and Baron-Cohen, 2002; Lutchmaya et al., 2002; Bayliss et al., 2005; Frischen et al., 2007). In conclusion, based on the current evidence we see little support for the joint line bisection task as a reliable overall measure of spatial allocation of social attention. Thus we cannot recommend it for future application within this domain. However, the data do suggest that should researchers wish to pursue the bisection task as a means for measuring social attention, we would encourage its investigation at the individual level, rather than the group level.

## Ethics Statement

The protocol was approved by the University of British Columbia Behavioural Research Ethics Board. All subjects gave written informed consent in accordance with the Declaration of Helsinki.

## Author Contributions

JD, AD, and AK conceived and designed the study. JD programmed the experiment and coordinated the acquisition of the data; wrote the first draft of the manuscript. JD and KR analyzed and interpreted the data. All authors contributed to the final manuscript.

## Conflict of Interest Statement

The authors declare that the research was conducted in the absence of any commercial or financial relationships that could be construed as a potential conflict of interest.

## Footnotes

1 After testing was completed, we noted that this instruction varied from Szpak et al. (2016), who asked participants to respond to the longer side of the line. However, our instruction is more consistent with the instruction sometimes used in the literature to judge whether the transector is to the left or right of the true line midpoint in the presence of a cue (McCourt and Olafson, 1997; Toba et al., 2011). Because task instructions may interact with performance on this type of task (Fink et al., 2002), we performed a control experiment to address the possibility that task instructions could yield a difference between our findings and those of Szpak and colleagues. Matching the main sample, we targeted a sample size of 32 participants after exclusions. Forty-seven new participants performed two blocks of the control task. In one block, participants followed the “respond shorter” instruction. In the other block, participants followed the “respond longer” instruction. Following the same exclusion criteria used for the main sample, 11 participants were excluded, leaving 36 for the analysis. Thresholds obtained in the two conditions were not statistically different [*t*(35) = -1.24, *p* = 0.22, BF_10_ = 0.36], indicating that the point of subjective equality measurement was not affected by instruction.
